# *In vitro *anti-inflammatory and anti-coagulant effects of antibiotics towards Platelet Activating Factor and thrombin

**DOI:** 10.1186/1476-9255-8-17

**Published:** 2011-07-07

**Authors:** Alexandros B Tsoupras, Maria Chini, Nickolaos Tsogas, Athina Lioni, George Tsekes, Constantinos A Demopoulos, Marios C Lazanas

**Affiliations:** 1 Faculty of Chemistry, National & Kapodistrian University of Athens, Panepistimioupolis of Zografou, Athens, 15771, Greece; 2 3rd Internal Medicine Dept.-Infectious Diseases Unit, Red Cross General Hospital, Athens, Greece

**Keywords:** Antibiotics, Lyso-PAF-AT, PAF, PAF-CPT, PAF-inhibitors, plasma-PAF-AH, Sepsis

## Abstract

**Background:**

Sepsis is characterized as a systemic inflammatory response that results from the inability of the immune system to limit bacterial spread during an ongoing infection. In this condition the significant mediator of inflammation Platelet Activating Factor (PAF) and the coagulant factor thrombin are implicated. In animal models, treatment with PAF-antagonists or co-administration of antibiotics with recombinant-PAF-Acetylhydrolase (rPAF-AH) have exhibited promising results. In order to examine the putative anti-inflammatory and/or antithrombotic interactions between antibiotic treatment used in sepsis with PAF and/or thrombin, we studied the *in vitro *effects of these compounds towards PAF or/and thrombin related activities and towards PAF basic metabolic enzymes.

**Methods:**

We assessed the inhibitory effect of these drugs against PAF or thrombin induced aggregation on washed rabbit platelets (WRPs) or rabbit Platelet Reach Plasma (rPRP) by evaluating their IC_50 _values. We also studied their effect on Cholinephosphotransferase of PAF (PAF-CPT)/Lyso-PAF-Acetyltransferase (Lyso-PAF-AT) of rabbit leukocytes (RLs), as well as on rabbit plasma-PAF-AH, the key enzymes of both *de novo*/remodelling PAF biosynthesis and PAF degradation, respectively.

**Results:**

Several antibiotics inhibited PAF-induced platelet aggregation of both WRPs and rPRP in a concentration-depended manner, with clarithromycin, azithromycin and amikacin exhibiting the higher inhibitory effect, while when combined they synergistically inhibited PAF. Higher concentrations of all antibiotics tested were needed in order to inhibit PAF induced aggregation of rPRP, but also to inhibit thrombin induced aggregation of WRPs. Concentrations of these drugs similar to their IC_50 _values against PAF activity in WRPs, inhibited also *in vitro *PAF-CPT and Lyso-PAF-AT activities of rabbit leukocytes, while only clarithromycin and azithromycin increased rabbit plasma-PAF-AH activity.

**Conclusions:**

These newly found properties of antibiotics used in sepsis suggest that apart from their general actions, these drugs may present additional beneficial anti-inflammatory and anti-coagulant effects against the onset and establishment of sepsis by inhibiting the PAF/PAF-receptor and/or the thrombin/protease-activated-receptor-1 systems, and/or by reducing PAF-levels through both PAF-biosynthesis inhibition and PAF-catabolism induction. These promising *in vitro *results need to be further studied and confirmed by *in vivo *tests, in order to optimize the efficacy of antibiotic treatment in sepsis.

## Background

Platelet Activating Factor (PAF) is a phospholipid signalling molecule of inflammation and a significant mediator of the immune system [1,2]. PAF transmits outside-in signals to intracellular transduction systems in a variety of cell types, including key cells of the innate immune and haemostatic systems: neutrophils, monocytes, and platelets [2]. Binding of PAF on specific membrane receptors coupled with G-proteins (PAF-receptor, PAFR) induces several intracellular signaling pathways that leads to auto/endo/para/juxta-crine cellular activation [3].

PAF can be synthesized by two different and distinct enzymatic routes, namely the remodeling and the *de novo* pathway [4-6]. The remodeling pathway involves a structural modification of 1-O-ether-linked membrane phospholipids where the action of cytoplasmic phospholipase A2 yields lyso-PAF which is then acetylated by a lyso-PAF:acetyl-CoA acetyltransferase (Lyso-PAF AT, EC 2.3.1.67) leading to the formation of PAF. In the *de novo* pathway, PAF-production occurs from simple molecules such as alkylglycerophosphate (AGP) in several steps. A central step is the conversion of 1-O-alkyl-2-acetyl-glycerol to PAF by a specific dithiothreitol-insensitive CDP-choline: 1-alkyl-2-acetyl-sn-glycerol cholinephosphotransferase (PAF-CPT, EC 2.7.8.16). Concerning PAF catabolism the most important enzyme involved is a PAF-specific acetylhydrolase (PAF-AH, EC 3.1.1.47), which cleaves the short acyl chain at the sn-2 position and forms lyso-PAF, which is biologically inactive [7].

Increased levels of PAF are implicated in several diseases, mainly inflammatory but also non-inflammatory ones [1-3], such as cardiovascular, renal and periodontal diseases [8-11], allergy [12], diabetes [13], cancer [14], AIDS [15] and Sepsis [16-23].

A great variety of molecules have been found to exhibit an inhibitory effect on PAF-induced biological activities, acting either through their direct antagonistic/competitive effect to PAF by binding on PAFR, or through other indirect mechanisms [24], that have not been fully clarified but seems to correlate with changes in the membrane microenvironment of PAF-receptor. Blockage of PAFR by such molecules represents a new therapeutic approach against several of the above mentioned diseases including Sepsis [16-23]. In addition, various PAF-inhibitors exhibit also the ability to *in vitro *and *in vivo *inhibit PAF-CPT, Lyso-PAF-AT and/or to induce PAF-AH activities [[15,25] unpublished data by AB Tsoupras).

Pharmacological data obtained with PAF antagonists, indicate a significant role for PAF in sepsis, septic shock and in the priming process [16-23]. Sepsis is a systemic inflammatory response that results from the inability of the immune system to limit bacterial spread during an ongoing infection. The effect of PAF antagonists in different models of sepsis and shock states indicates a role for PAF in endotoxin associated lethality, activation of inflammatory blood cells with release of mediators, cardiovascular failure and increased vascular permeability, as well as in the development of shock organs and organ failure.

The precise role of PAF as mediator of the diffuse inflammatory state characteristic of sepsis remains to be determined, but, in animal models, beneficial effects have been observed as a result of treatment with various antagonists of PAF [16-23]. Strategies to block inflammatory mediators such as PAF, often with complicated outcomes, are currently being investigated as new adjuvant therapies for sepsis. To date, however, it has been impossible to duplicate these encouraging results from animal models in the clinical setting.

On the other hand, administration of recombinant PAF-AH (rPAF-AH), protects mice from inflammatory injury and death after administration of lipopolysaccharide (LPS) or cecal ligation and puncture (CLP) [26]. Co-administration of antibiotics together with rPAF-AH was more effective than single treatment with either of these agents [26]. The beneficial effects of this combined treatment suggest a potential role of antibiotics against PAF implication in sepsis.

In order to examine the possible interactions between PAF and antibiotic treatment against sepsis we studied their potential effect on PAF-metabolism and/or their putative anti-PAF activity.

For this reason in the present study we examined for the first time the *in vitro *anti-inflammatory and anti-thrombotic ability of a broad-spectrum of antibiotics and several of their combinations/regimens used in treatment against sepsis, based on their effect towards PAF-induced or thrombin induced platelet aggregation of Washed Rabbit Platelets (WRPs) and rabbit Platelet Reach Plasma (rPRP). In addition we examined their ability to affect PAF-metabolism by decreasing PAF-activity, through their *in vitro *effect on PAF basic metabolic enzymes, PAF-CPT and lyso PAF-AT of rabbit leukocytes as well as rabbit plasma PAF-AH.

## Materials and methods

### Materials and instruments

Centrifugations were performed in an Heraeus Labofug 400R and a Sorvall RC-5B refrigerated super speed centrifuge Homogenizations were conducted in a supersonic sonicator (Sonics & Materials, Newtown, CT, USA). The liquid scintillation counter used was a 1209 Rackbeta (Pharmacia, Wallac, Finland). PAF-induced platelet aggregation studies were performed in a model 400 VS aggregometer of Chrono-Log (Havertown, PA, USA) coupled to a Chrono-Log recorder at 37°C with constant stirring at 1200 rpm.

BSA (bovine serum albumin), PAF (1-O-hexadecyl-2-acetyl-sn-glycero-3-phosphocholine), thrombin, trichloroacetic acid (TCA), CDP-choline, lyso-PAF, acetyl-CoA, dithiothreitol (DTT), EDTA, MgCL_2_, Tris and analytical reagents and solvents were purchased from Sigma (St.Louis, MO, USA). 1-O-hexadecyl-2-[^3^H]acetyl-sn-glycerol-3-phosphocholine ([^3^H] PAF) with a specific activity of 10 Ci/mmol was obtained from New England Nuclear (Dupont, Boston, MA, USA). 1-O-alkyl-2-sn-acetyl-glycerol (AAG) was purchased from BIOMOL International LP (Palatine House, Matford Court, Exeter, UK). 2,5-Diphenyloxazole (PPO) and 1,4-bis(5-phenyl-2-oxazolyl) benzene (POPOP) were purchased from BDH Chemicals (Dorset, England). Scintillation liquid cocktail (dioxane base) was prepared by diluting 7 g PPO, 0.3 g POPOP and 100 g Napthalene in 200 mL H_2_O and then transferred to 1 L of dioxane. Liquid chromatography grade solvents and silica G for TLC were purchased from Merck KGaA (Darmstadt, Germany).

The antibiotics that were tested were provided by our hospital pharmacy and were dissolved in 2.5 mg bovine serum albumin (BSA)/mL saline [1,15]. In order to test several combinations of antibiotic regimens, several mixtures of these drugs were also prepared using the above solutions of each drug. The ratios of the concentrations (μg/μL) of the active components that were used in each mixture are shown in Table two.

### Biological assays on Washed Rabbit Platelets (WRPs) and rabbit Platelet Reach Plasma (rPRP)

We assessed the *in vitro *inhibitory effect of these drugs and their combinations in anti-septic treatment regimens against PAF-induced or thrombin induced aggregation on WRPs and rPRP by evaluating the concentration (μg/mL) of the bioactive compound(s) in each case in the aggregometer cuvette that inhibited 50% PAF-induced or thrombin induced aggregation (IC_50_) of WRPs or rPRP, as previously described [1,15,27,28]. Briefly, PAF and the examined drugs were dissolved in 2.5 mg BSA/ml saline. The drugs were tested for their ability to inhibit PAF-induced aggregation of WRPs or rPRP and thrombin induced aggregation of WRPs and/or to induce WRPs aggregation in a Chrono-Log aggregometer. Various concentrations of the examined samples were added into the aggregometer cuvette 1 min prior to the addition of PAF or thrombin. The platelet aggregation induced by PAF (4.4 × 10^-11 ^M and 2.24 × 10^-7^M, final concentration in the aggregometer cuvette in the cases of WRPs and rPRP respectively) or thrombin (0.01 mU in the aggregometer cuvette in the case of WRPs) was measured as PAF-induced or thrombin induced aggregation in WRPs or rPRP before (considered as 0% inhibition) and after the addition of various concentrations of the examined sample [15,27,28]. A linear plot of inhibition percentage (ranging from 20% to 80%) versus the concentration of the sample was established for each antibiotic and in each case. From this curve, the concentration of the sample that inhibited 50% of the PAF or thrombin induced aggregation (IC_50_) was calculated. The aggregatory activity of the sample was expressed as micrograms of the bioactive compound(s) of the drugs dissolved in 2.5 mg BSA/ml saline, which is able to induce 50% of the maximum reversible aggregation of the respective sample, defined as EC_50 _value. In addition, desensitization tests were carried out as previously described [15,27]. Briefly, in desensitization and cross-desensitization experiments, platelets were activated by the addition of PAF or drugs to the platelet suspension at a concentration that caused reversible aggregation. Second stimulation with the tested bioactive compound(s) or PAF respectively, was performed immediately after complete disaggregation.

### Isolation of plasma and leukocytes from rabbit blood

The isolation of plasma and leukocytes from rabbit blood was performed as previously described [15] with some modifications. Briefly: 9 mL of blood were obtained from each rabbit in 1 mL of an anticoagulant solution of sodium citrate/citrate acid.

The sample was centrifuged at 630 g for 10 min at 25°C (1st centrifugation). The supernatant (plasma reach in platelets) was centrifuged at 1400 g for 20 min at 25°C (2nd centrifugation).

The supernatant of the 2nd centrifugation (plasma) was aliquoted and stored at -80°C until the time of the plasma PAF-AH assay analysis.

From the pellet of the 1st centrifugation (leukocytes and erythrocytes) the isolation of the leukocytes from the contaminating erythrocytes was achieved by erythrocyte sedimentation. Saline was added in order the sample reached its initial volume of 10 mL. The sample was separated in half and 1.7 mL of dextran solution (3% dextran in NaCl 0.15 M) was added in each half and the mixtures were kept for 1 h at room temperature. The leukocyte-rich supernatants were then centrifuged at 500 g for 10 min at room temperature (4th centrifugation). Contaminating erythrocytes of the sediment were lysed with the addition of a lysis solution consisting of 155 mM NH_4_Cl, 10 mM KHCO_3_, and 0.1 mM EDTA and then removed with a centrifugation at 300 g for10 min at room temperature (5th centrifugation).

The pelleted cells of the 5th centrifugation (isolated leukocytes) were resuspended in 1 ml of a buffer containing 50 mM Tris-HCl (pH 7.4) and sonicated on ice (4 × 15 s). Then they were centrifuged at 500 g for 10 min at 4°C (6th centrifugation) in order to remove whole cells, nucleuses and debris in the pellet and the supernatants (homogenates) after protein determination were aliquoted and stored at -80°C until the time of the PAF-CPT and Lyso-PAF-AT assays analysis.

### DTT-insensitive PAF-Cholinephosphotransferase (PAF-CPT) activity assays

Assay was performed on the homogenates of rabbit leukocytes as previously described [15,25]. Briefly, the reaction was carried out at 37°C for 20 min in a final volume of 200 μL containing 0.05-2.5 mg/mL protein, 100 mM Tris-HCl (pH 8.0), 15 mM dithiothreitol (DTT), 0.5 mM EDTA, 20 mM MgCl_2_, 1 mg/mL BSA, 100 μM CDP-Choline and 100 μΜ 1-O-alkyl-2-sn-acetyl-glycerol (AAG). The reaction was stopped by adding 0,5 ml of cold methanol (2% acetic acid). The extraction, purification and determination of PAF were performed as previously described [25]. Briefly, 0,25 ml of cold chloroform was added in order to firstly reach the proportion of 1/2/0.8 CHCl_3_:MeOH:H_2_O, and after potent vortex another 0,25 ml of cold chloroform and 0,25 ml of water were added in order to finally reach the proportion of 1/1/0.9 CHCl_3_:MeOH:H_2_O from where produced PAF was extracted in the chloroform phase by the acid Bligh-Dyer method [29]. The extracted PAF was further separated by thin-layer chromatography (TLC) on Silica Gel G coated plates with an elution system consisting of chloroform:methanol:acetic acid: water (100:57:16:8, v/v/v/v). The band corresponding to PAF (between lyso-phosphatidylcholine and phosphatidylcholine) was identified by co-chromatographing lipid standards which were visualized by exposure of the plates to iodine vapors. PAF fractions were scrapped off, extracted by the Bligh-Dyer method [29] and the amount of PAF was determined by the washed rabbit platelet aggregation assay [1]. All assays were performed in duplicate. Enzymatic activities were expressed as specific activity in nmol/min/mg of total protein.

The effect of drugs on PAF-CPT activity was evaluated in homogenates of rabbit leukocytes. The *in vitro *enzymatic assay of PAF-CPT was performed in the presence of several concentrations of each drug in the assay reaction mixture as previously described [15].

### Lyso-PAF-AT activity assays

Assay was performed on the homogenates of leukocytes as previously described [15]. Briefly, the reaction was carried out at 37°C for 30 min in a final volume of 200 μL containing 0.05-2.5 mg/mL protein, 50 mM Tris-HCl (pH 7,4), 0.25 mg/mL BSA, 20 μΜ Lyso-PAF and 200 μΜ acetyl-CoA. The reaction was stopped by adding 2% acetic acid methanol and the extraction, purification and determination of PAF was carried out as mentioned above in the PAF-CPT-assay [25]. All assays were performed in duplicate. Enzymatic activities were expressed as specific activity in nmol/min/mg of total protein.

The effect of drugs on Lyso-PAF-AT activity was also evaluated in homogenates of rabbit leukocytes. The *in vitro *enzymatic assay of Lyso-PAF-AT was performed in the presence of several concentrations of each drug in the assay reaction mixture as previously described [15].

### Plasma PAF-AH activity assays

Plasma-PAF-AH activity was determined by the trichloroacetic acid precipitation method using [^3^H]-PAF as a substrate as previously described [30]. Briefly, 2 μL of plasma were incubated with 4 nmol of [^3^H] PAF (20 Bq per nmol) for 30 min at 37°C in a final volume of 200 μL of 50 mM Tris/HCl buffer (pH 7.4). The reaction was terminated by the addition of cold trichloroacetic acid (10% final concentration). The samples were then placed in an ice bath for 30 min and subsequently centrifuged at 16000 g for 5 min. The [^3^H]-acetate released into the aqueous phase was measured on a liquid scintillation counter. All assays were performed in duplicate. The enzyme activity was expressed as nmol of PAF degraded per min per mL of plasma.

The effect of drugs on PAF-AH activity was evaluated in rabbit plasma. The *in vitro *enzymatic assay of plasma PAF-AH was performed in the presence of several concentrations of each drug in the assay reaction mixture as previously described [15].

#### Analytical methods

Protein concentrations, determined according to the method of Bradford [31], were based on BSA as the protein standard.

#### Statistical analysis

Normal distribution of variables was checked using Kolmogorov-Smirnov criterion before further analyses. Data are expressed as geometrical mean with 95% confidence limits along with median, minimum and maximum values for IC50 values and as mean values ± SD for enzyme activities. Differences in PAF-metabolic enzymes activities in the presence and in the absence (control) of drugs were assessed by multiple comparisons with one way ANOVA using LSD post-hoc tests and were considered to be statistically significant when p < 0.05. Data were analyzed using a statistical software package, SPSS 18.0, and Microsoft Excel 2007 for Windows.

## Results

Several antibiotics inhibited *in vitro *PAF induced aggregation of washed rabbit platelets in a concentration-dependant manner. Their IC_50 _values against PAF are expressed as micrograms/mL (μg/mL) of bioactive compound in the aggregometer cuvette that cause 50% inhibition of PAF-induced washed rabbit platelet aggregation in a final concentration of 4.4 × 10^-11 ^M (Table 1). The IC_50 _values ranged from 0.19 to 110.95 μg/mL, approximately. The most potent ones in the rank were clarithromycin, azithromycin, linezolid, amicacin and netilmycin. Other drugs studied such as meropenem and vancomycin, did not influence PAF activity in WRPs.

**Table 1:** *In vitro *inhibitory effect (expressed as IC_50_) of the antibiotics tested against PAF-induced aggregation of WRPs and their ability to induce platelet aggregation

	**IC**_**50**_^**1 **^**towards PAF in WRPs (μg/mL)**					
Bioactive Compound	Median	Min	Max	Geometric Mean	95% Confidence Interval	Drug-induced WRPs aggregation/desensitization
Clarithromycin	0.18	0.14	0.28	0.19	0.08 thru 0.46	-/-
Azithromycin	0.40	0.20	0.85	0.41	0.07 thru 2.46	-/-
Linezolid	1.25	0.60	1.62	1.07	0.30 thru 3.84	-/-
Amikacin	2.73	1.50	4.55	2.65	0.67 thru 10.54	-/-
Netilmicin	2.80	1.45	4.70	2.67	0.62 thru 11.56	-/-
Daptomycin	5.01	2.88	7.22	4.71	1.49 thru 14.85	-/-
Piperacillin/Tazobactam	17.65/2.22	12.18/1.54	22.27/2.85	16.85/2.14	7.91 thru 35.90/0.99 thru 4.61	-/-
Ceftazidime	30.06	20.92	37.95	28.79	13.66 thru 60.68	-/-
Tigecycline	113.45	91.86	131.07	110.95	71.16 thru 173.0	-/-
Vancomycin	ND	-	-	-	-	+/-
Meropenem	ND	-	-	-	-	-/-

Experiments were conducted three times using different platelets preparations. ^1^IC_50_ values are expressed as μg/mL of bioactive compound in the aggregometer cuvette, Final concentration of PAF in the aggregometer cuvette when tested in WRPs was 4.4 × 10^-11^M. WRPs: Washed Rabbit Platelets; ND: Not detected inhibition against PAF-induced platelet aggregation; -/-: Not detected platelet aggregation; +/-: Detected platelet aggregation/not detected platelet desensitization against PAF.

From all antibiotics tested, only vancomycin, induced aggregation in WRPs in a concentration much higher than its IC_50 _value (Table 1). Desensitization and cross desensitization experiments showed that vancomycin seemed to induce platelet aggregation through a different way than that of PAF pathway (Table 1).

All antibiotics were further tested for their potential inhibitory effect against the PAF-induced rabbit PRP aggregation. Their IC_50 _values in this case are also expressed as micrograms/mL (μg/mL) of bioactive compound in the aggregometer cuvette that cause 50% inhibition of PAF-induced aggregation of rPRP in a final concentration of 2.24 × 10^-7 ^M (Table 2). These IC_50 _values ranged from 8.3 to 829.0 μg/mL, approximately. In the case of rPRP the most potent antibiotics in the rank were amicacin, azithromycin, tygecycline and clarithromycin, while other drugs studied such as meropenem and linezolid, did not influence PAF activity in rPRP.

**Table 2:** *In vitro *inhibitory effect (expressed as IC_50_) of the antibiotics tested against PAF-induced aggregation of rPRP

	**IC**_**50**_^**1 **^**towards PAF in rPRP (μg/mL)**				
Bioactive Compound	Median	Min	Max	Geometric Mean	95% Confidence Interval
Clarithromycin	49.6	33.2	78.4	50.5	17.4 thru 147.1
Azithromycin	23.3	11.9	29.6	20.2	6.2 thru 65.2
Linezolid	ND	-	-	-	-
Amikacin	9.6	5.4	11.2	8.3	3.2 thru 21.7
Netilmicin	384.6	365.9	430.4	392.7	319.4 thru 482.9
Daptomycin	384.5	375.8	465.8	406.8	303.5 thru 545.2
Piperacillin/Tazobactam	837.1/86.4	765.0/76.9	889.6/102.3	829.0/87.9	686.5 thru 1001.0/61.6 thru 125.6
Ceftazidime	385.5	345.6	412.9	380.3	304.3 thru 475.3
Tigecycline	26.0	20.8	27.3	24.6	17.1 thru 35.2
Vancomycin	70.9	62.1	73.7	68.7	55.0 thru 86.0
Meropenem	ND	-	-	-	-

Experiments were conducted three times using different platelets preparations. ^1^IC_50_ values are expressed as μg/mL of bioactive compound in the aggregometer cuvette. Final concentration of PAF in the aggregometer cuvette when tested when tested in rPRP was 2.24 × 10^-7^M. rPRP: rabbit Platelet Reach Plasma; ND: Not detected inhibition against PAF-induced platelet aggregation.

All antibiotics were also tested for their potential inhibitory effect towards the thrombin induced WRP's aggregation. Their IC_50 _values in this case are also expressed as micrograms/mL (μg/mL) of bioactive compound in the aggregometer cuvette that cause 50% inhibition of thrombin-induced aggregation of WRPs in a final concentration of 0.01 mU (Table 3). These IC_50 _values ranged from 6.7 to 350.3 μg/mL, approximately. In this case, the most potent antibiotics in the rank were netilmicin, azithromycin, amicacin and daptomycin, while again meropenem did not influence thrombin activity in WRPs.

**Table 3:** *In vitro *inhibitory effect (expressed as IC_50_) of the antibiotics tested against thrombin induced aggregation of WRPs

	**IC**_**50**_^**1 **^**towards Thrombin in WRPs (μg/mL)**				
Bioactive Compound	Median	Min	Max	Geometric Mean	95% Confidence Interval
Clarithromycin	105.6	88.3	119.5	103.7	71.0 thru 151.3
Azithromycin	13.6	11.9	14.5	13.3	10.3 thru 17.1
Linezolid	98.0	93.0	110.1	100.1	80.8 thru 124.1
Amikacin	22.0	18.7	27.3	22.4	14.0 thru 36.0
Netilmicin	6.6	5.7	8.1	6.7	4.3 thru 10.4
Daptomycin	42.7	33.7	45.9	40.4	27.1 thru 60.4
Piperacillin/Tazobactam	142.3/17.8	123.6/15.6	170.1/20.8	144.1/17.9	96.8 thru 214.4/12.5 thru 25.7
Ceftazidime	99.2	82.8	115.3	98.2	65.0 thru 148.3
Tigecycline	262.0	222.7	311.6	262.9	173.2 thru 399.1
Vancomycin	354.0	312.7	388.5	350.3	267.3 thru 459.2
Meropenem	ND	-	-	-	-

Experiments were conducted three times using different platelets preparations. ^1^IC_50_ values are expressed as μg/mL of bioactive compound in the aggregometer cuvette. Final concentration of thrombin in the aggregometer cuvette was 0.01 mU in WRPs. WRPs: Washed Rabbit Platelets; ND: Not detected inhibition against thrombin-induced platelet aggregation.

Several combinations of these drugs were also tested against PAF-induced aggregation of WRPs. The most potent ones are presented in Table 4. Among the combinations of antibiotics of regimens against sepsis that were tested, piperacillin-tazobactam/netilmicin, piperacillin-tazobactam/amikacin, ceftazidime/amikacin, ceftazidime/netilmicin displayed the higher inhibitory effect against PAF activity in WRPs, respectively (Table 4).

**Table 4:** *In vitro *inhibitory effect (expressed as IC_50_) of the most potent combinations of antibiotic anti-septic regimens against PAF-induced WRPs aggregation

		**IC**_**50**_^**2 **^**towards PAF in WRPs (μg/mL)**				
Combinations of Bioactive Compounds	**Ratio**^**1**^	Median	Min	Max	Geometric Mean	95% Confidence Interval
Piperacillin-Tazobactam/Netilmicin	40-5/1	5.1-0.6/0.1	4.6-0.6/0.1	5.3-0.7/0.1	5.0-0.6/0.1	4.2 thru 6.0-0.5 thru 0.8/0.1 thru 0.1
Piperacillin-Tazobactam/Amikacin	40-5/1.7	5.1-0.6/0.2	4.6-0.6/0.2	5.4-0.7/0.2	5.1-0.6/0.2	4.2 thru 6.1-0.5 thru 0.8/0.2 thru 0.2
Ceftazidime/Amikacin	6/1	10.0/1.7	7.4/1.2	13.1/2.6	9.9/1.7	4.9 thru 20.1/0.7 thru 4.6
Ceftazidime/Netilmicin	10/1	10.6/1.1	7.8/0.8	14.3/1.4	10.6/1.1	5.0 thru 22.5/0.5 thru 2.2
Meropenem/Netilmicin	10/1	15.3/1.5	12.2/1.2	21.4/2.1	15.9/1.6	7.9 thru 32.0/0.8 thru 3.1
Meropenem/Amikacin	6/1	22.5/3.8	18.8/3.1	31.9/5.3	23.8/4.0	12.2 thru 46.4/2.0 thru 7.8

Experiments were conducted three times using different platelets preparations. ^1^Ratio of concentrations of bioactive compounds in each mixture. ^2^IC_50 _values are expressed as μg/mL of each antibiotic in the mixture that was added in the aggregometre cuvette. Final concentration of PAF in the aggregometer cuvette when tested in WRPs was 4.4 × 10^-11^M. WRPs: Washed Rabbit Platelets.

**Figure 1:**
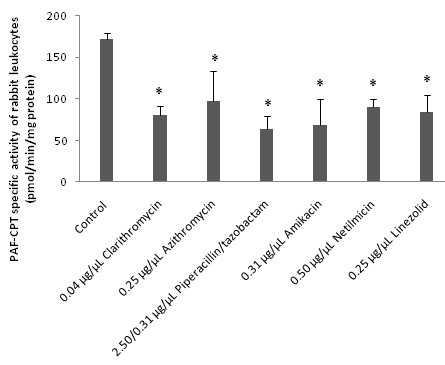
***In vitro *inhibitory effect of antibiotics towards PAF-CPT enzyme activity of rabbit leukocytes**. The amounts of each drug that induced approximately fifty to one hundred inhibitory effects against PAF-CPT specific activity are expressed as μg of each bioactive compound added in the assay mixture/μL of assay volume. PAF-CPT specific activity of rabbit leukocytes is expressed as nmol of produced PAF/min/mg of total protein in assay. Control signifies PAF-CPT specific activity of rabbit leukocytes in the absence of any drug. Results are the average of three independent determinations using different enzyme preparations performing duplicate samples. (* p < 0.05 compared to control). PAF-CPT: Cholinephosphotransferase of PAF.

**Figure 2:**
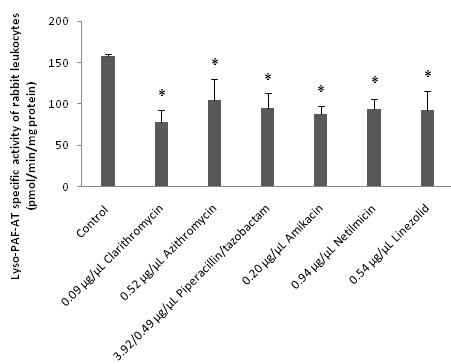
***In vitro *inhibitory effect of antibiotics towards Lyso-PAF-AT enzyme activity of rabbit leukocytes**. The amounts of each drug that induced approximately fifty to one hundred inhibitory effects against Lyso-PAF-AT specific activity are expressed as μg of each bioactive compound added in the assay mixture/μL of assay volume. Lyso-PAF-AT specific activity of rabbit leukocytes is expressed as nmol of produced PAF/min/mg of total protein in assay. Control signifies Lyso-PAF-AT specific activity of rabbit leukocytes in the absence of any drug. Results are the average of three independent determinations using different enzyme preparations performing duplicate samples. (* p < 0.05 compared to control).

In addition when these drugs were added, in concentrations similar to their IC_50 _values against PAF activity, in the enzymatic assays of both PAF-CPT and Lyso-PAF-AT of rabbit leukocytes they *in vitro *significantly inhibited both enzymes activities in a concentration depended manner (p < 0.05 in relevance to control assays). In Figures 1 and 2 are shown the amounts of each drug that induced approximately fifty to one hundred inhibitory effect against PAF-CPT and Lyso-PAF-AT specific activities respectively (Figures 1, 2). Moreover, the amount of clarithromycin needed in order to achieve this inhibition in both PAF-CPT and Lyso-PAF-AT was found one order of magnitude lower than those of all the other antibiotics, with the exception of that of amikacin in the case of Lyso-PAF-AT inhibition, which in turn was also much lower than those of all the other antibiotics tested.

**Figure 3:**
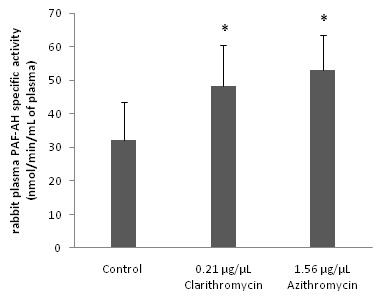
***In vitro *effect of antibiotics towards rabbit plasma PAF-AH enzyme activity**. The amounts of each drug that induced significant increase on specific activity are expressed as μg of each bioactive compound added in the assay mixture/μL of assay volume (p < 0.05 versus control). Rabbit plasma PAF-AH specific activity is expressed as nmol of degraded PAF/min/mg of total protein in assay. Control signifies rabbit plasma PAF-AH specific activity of rabbit leukocytes in the absence of any drug. Results are the average of three independent determinations using different enzyme preparations performing duplicate samples. (* p < 0.05 compared to control). Plasma PAF-AH: plasma PAF-Acetylhydrolase

On the other hand, from the entire drug tested only clarithromycin and azithromycin induced an *in vitro *significant increase of rabbit plasma PAF-AH (p < 0.05 in relevance to control assays), in concentrations of an order of magnitude higher than their IC_50 _values against PAF. In Figure 3 are shown the amounts of these two drugs that induced the significant increase of rabbit plasma PAF-AH enzyme activity (Figure 3). Moreover, the amount of clarithromycin needed in order to achieve this induction in plasma-PAF-AH was found one order of magnitude lower than that of azithromycin (p < 0.05)

## Discussion

Sepsis is a systemic inflammatory response that results from the inability of the immune system to limit bacterial spread during an ongoing infection. The pathophysiology of sepsis is not completely understood. Bacteria are the main cause of sepsis. Activated receptors of the innate immune system lead to an exaggerated immune response including systemic inflammation. Immune cells including activated neutrophils and macrophages express and are controlled by a variety of cytokines, chemokines, complement factors and other mediators such as PAF and Thrombin [16-23,32]. The activation of toll-like receptors such as TLR4 usually leads to further amplification of inflammation through these mediators [32]. These receptors have been found to be directly activated by bacteria Lipopolysaccharide (LPS) and thus inducing PAF biosynthesis by the phosphorylation and subsequently activation of Lyso-PAF-AT enzyme activity [33].

Both PAF and thrombin are implicated in severe inflammatory and coagulant procedures occurring during sepsis [24,32]. In addition it has been recently proposed that in chronic pathological states such as in cancers like melanoma, the PAF- and thrombin-activated pathways are interrelated, thus regulating, for instance, both the melanoma cell adhesion and its metastasis [34,35]. Critically ill patients often have systemic activation of both inflammation and coagulation [32]. Increasing evidence points to an extensive cross-talk between these two systems, whereby inflammation not only leads to activation of coagulation, but coagulation also considerably affects inflammatory activity [32]. The intricate relationship between inflammation and coagulation may have major consequences for the pathogenesis of microvascular failure and subsequent multiple organ failure, as a result of severe infection and the associated systemic inflammatory response.

Beneficial effects have been observed as a result of treatment with various inhibitors or antagonists of PAF in different shock states and animal models [16-23]. To date, however, it has been impossible to translate these encouraging results from animal models in the clinical setting.

Recent studies in the field of gaining beneficial and promising results from an anti-PAF approach in several diseases have been focused in an effort not only to inhibit PAF action but also to down regulate its levels, through the inhibition of its biosynthesis and/or induction of its degradation [14,15,25,26]. For example, administration of rPAF-AH, protects mice from inflammatory injury and death after administration of lipopolysaccharide (LPS) or cecal ligation and puncture (CLP) [26]. Co-administration of antibiotics together with rPAF-AH was more protective than single treatment with either of these agents [26].

To our knowledge there are no other studies on the possible anti-inflammatory and anti-thrombotic properties of antibiotics used in sepsis treatment through their anti-PAF or anti-thrombin activities. This is the first study to report the anti-inflammatory and anti-thrombotic activities of a wide spectrum of antibiotics through their effects on PAF biological activities and its metabolism, as well as on thrombin. We also studied the effect of several of their combinations of treatment regimens in sepsis, against PAF activity.

In this study, in the case of the anti-PAF activities of the antibiotics tested, the biological assays were focused on the PAF-induced aggregation of both WRP's and rabbit PRP. In particular, our study on WRPs probes the anti-PAF activity of antibiotics under the experimental conditions applied, while, in the case of rabbit PRP, the conclusions drawn pinpoint the effect of these compounds on the PAF activation, similar to the *in vivo* conditions. In addition, the IC_50 _values measured in each case reflect the inhibition strength of each antibiotic, since a low IC_50 _value reveals stronger inhibition of the PAF-induced aggregation of either WRPs or rPRP for a given antibiotic concentration.

Our work leads to the conclusion that apart from their general anti-septic actions several antibiotics exhibit also a potent *in vitro *inhibitory effect against PAF-induced aggregation of both WRPs and rPRP, in a dose-dependent manner (Tables 1 and 2). Significantly higher concentrations (at least one order of magnitude) of each compound were needed in order to inhibit the PAF-induced aggregation of rabbit PRP, compared to those needed in order to inhibit the corresponding aggregation of WRPs.

In the case of WRPs the antibiotics with the most prominent anti-PAF activity were clarithromycin, azithromycin, linezolid, amikacin and netilmicin, while in the case of rPRP were amikacin, azithromycin, tigecycline and clarithromycin. These results suggest that from all antibiotics tested in both WRPs and rPRP, the same three amikacin, azithromycin and clarithromycin, belonged to the ones with the most potent anti-PAF effect, even though higher concentrations of these drugs were needed in the case of rPRP. Only in the case of amikacin its IC_50 _values towards PAF-induced aggregation of both WRPs and rPRP were at the same order of magnitude.

Furthermore, tigecycline with one of the lowest anti-PAF effects in WRPs exhibited a potent anti-PAF effect in the case of rPRP; only in this antibiotic its IC_50 _value towards PAF-induced aggregation of rPRP was approximately 5 times lower than that towards PAF-induced aggregation of WRPs. On the other hand, in the cases of linezolid and netilmicin with potent anti-PAF effects in WRPs, the first antibiotic did not inhibited PAF-induced aggregation of rPRP at all, while the second one exhibited one of the lowest anti-PAF effects in this case.

However, some of these drugs such as meropenem and vancomycin, did not influence PAF activity in WRPs, while the first one did not also inhibited PAF-induced aggregation of rPRP at all. Moreover, vancomycin induced *in vitro *aggregation of washed rabbit platelets, while cross-desensitization experiments showed that this platelet activation seems to take place through a different way than that of PAF-PAFR pathway.

It should also be noted that the anti-PAF activity of these drugs in WRPs was found similar to the most potent of other antimicrobial drugs that have been recently found to exhibit anti-PAF activity [15]. The IC_50 _values of these antibiotics against PAF share same or slightly less order of magnitude in comparison with the relatively IC_50 _values of some of the most potent PAF receptor-specific antagonists used in several models against sepsis and other diseases, such as WEB2170, BN52021, and rupatadine [18,36,37] (0.009, 0.013, and 0.106 μg/mL in the aggregometer cuvette, respectively).

Moreover, some of these drugs seem to act synergistically against PAF-induced platelet aggregation in some but not in all combinations of treatment regimens against sepsis that were tested (Table 4). For example, when ceftazidime with the one of the lowest anti-PAF activity in WRPs (IC_50 _= 28.79 μg/mL) was combined with either netilmicin with an IC_50 _value of 2.67 μg/mL or with amikacin with an IC_50 _value of 2.65 μg/mL, the final mixture inhibited PAF-induced platelet aggregation with IC_50 _values of 10.6/1.1 μg/mL of the first or 9.9/1.7 μg/mL of the second mixture in the aggregometer cuvette respectively (Table 4). The synergistic anti-PAF action of these antibiotics when combined seems to belong to a more general pattern, since other antimicrobial drugs also when combined have been found to synergistically inhibit PAF [15]. It should be noted that the selection of antibiotic regimens tested was based on doses of these drugs that are usually administrated in patients, as well as from the IC_50 _values of each drug against PAF activity.

All antibiotics were additionally tested on the thrombin induced aggregation of WRPs. In the present study we have found also for the first time that several of these antibiotics exhibit additionally anti-thrombotic properties by inhibiting thrombin induced aggregation of WRPs in a concentration depended manner (Table 3). The antibiotics with the most prominent anti-thrombin activity were netilmicin and again azithromycin and amikacin. However, significantly higher concentrations (at least one order of magnitude, with the exception of netilmicin) of each compound were needed in order to inhibit the thrombin-induced aggregation of WRPs, compared to those needed in order to inhibit the corresponding PAF-induced aggregation of WRPs (Tables 1 and 2). This result points out that WRPs were actually viable and still normally functioning after incubation with concentrations of these antibiotics near their IC_50 _values towards PAF under the experimental conditions used, given that when platelets were incubated with much higher concentrations of these drugs they were aggregated normally when thrombin was used (in concentrations lower than their IC_50 _values towards thrombin).

In addition since much higher concentrations of these antibiotics were needed in order to 50% inhibit thrombin in WRPs, it seems that these drugs exhibit a more general anti-inflammatory action, which, however, is more specific towards the PAF-related pathway. Only in the case of netilmicin its IC_50 _value towards thrombin was in the same order of magnitude with that towards PAF; approximately 2 folds higher than that towards PAF. As a result this antibiotic exhibited the most potent inhibition towards thrombin, suggesting that netilmicin exhibits a more general anti-inflammatory and anti-thrombotic activity, since it can inhibit both the PAF and thrombin-related activities in concentrations in the same order of magnitude.

Taking into account all the above, one may suggest that apart from their general activities including their beneficial effects in sepsis, some of these drugs exhibit also a remarkable *in vitro *inhibitory effect against PAF or thrombin activities, while others did not affect PAF or thrombin activities, implying different perspectives for each antibiotic towards inflammatory and coagulant manifestations that usually occur during sepsis [21,32]. The observed differences between all drugs' inhibitory effects towards PAF and thrombin activities in different platelet preparations, WRPs and rPRP, point out dissimilar anti-inflammatory and/or anti-thrombotic potentials for each antibiotic and may be related to differences in their chemical structures and/or in their interactions with cell-membranes and/or plasma constituents.

Furthermore, in order to determine the possible interactions between these drugs and PAF metabolism, the *in vitro *effect of some of these drugs on the activities of PAF metabolic enzymes PAF-CPT, Lyso-PAF-AT and PAF-AH was also studied. For this purpose, we evaluated the specific activities of PAF-CPT and Lyso-PAF-AT of homogenates of rabbit leukocytes, as well as rabbit plasma PAF-AH in the presence of each antibiotic in the assay mixture. We found for the first time that several of the antibiotics tested inhibited *in vitro *both PAF biosynthetic enzymes in a concentration depended manner (Figures 1 and 2), while only clarithromycin and azithromycin induced an *in vitro *increase of rabbit plasma PAF-AH, in concentrations an order of magnitude higher than those of PAF-biosynthesis inhibition and their IC_50 _values against PAF (Figure 3).

Smaller amounts (one to two order of magnitude) of clarithromycin were needed in order to fifty to one hundred inhibit PAF-CPT and Lyso-PAF-AT specific activities, in relevance to the other drugs tested. This result, aided by the facts that this antibiotic seems to induce PAF-degradation in lower concentrations than the other antibiotics tested and potently inhibit PAF-induced platelet aggregation, propose a promising role for this drug as far as concerns its potent anti-inflammatory activity in sepsis.

Moreover, the amounts of all antibiotics that were needed in order to fifty to one hundred inhibit Lyso-PAF-AT specific activity were twice higher than those for the relevant inhibition of PAF-CPT, except for amikacin, where lesser amounts were needed. This result may be a sign of irreconcilable differences in the inhibitory effect of these antibiotics against the two distinct biosynthetic routes of PAF. Taking also into account that amikacin exhibited one of the most potent anti-PAF effects (this antibiotic was the only one that its low IC_50 _values were in the same order of magnitude towards PAF-induced aggregations of both WRPs and rPRP) and one of the most potent anti-thrombotic effects, the additional potent inhibitory effect of this antibiotic towards PAF-biosynthesis provide new anti-inflammatory potentials for this drug.

Taking into account that during sepsis PAF synthesis is induced by bacteria LPS through toll-like receptors [33], the inhibitory effect of some of these drugs against PAF biosynthetic enzymes may reduce PAF-synthesis, down regulating thus PAF-activity and subsequently PAF-related inflammatory procedures.

## Conclusions

This is the first study to bring in surface putative anti-inflammatory and anti-thrombotic activities of some antibiotics used in sepsis, through their *in vitro *studied anti-PAF and anti-thrombin effects in rabbit platelets. Furthermore, these drugs have exhibited the ability to inhibit also PAF-synthesis. Amicacin, clarithromycin and azithromycin with the most potent anti-PAF activities in both WRPs and rPRP, showed the most potent inhibitory effect also towards PAF-biosynthesis, while clarithromycin and azithromycin were the only ones that could induce PAF-degradation. Amikacin also inhibited potently thrombin.

It seems that these newly found anti-inflammatory and anti-thrombotic properties of antibiotics and/or antibiotic regimens used in sepsis, such as their inhibitory activities towards PAF/PAFR and thrombin pathways, as well as their interactions with PAF-metabolism, may provide new perspectives for these drugs towards also the inflammatory and coagulant manifestations that usually take place during several septic stages, including induced by severe sepsis multiple organ failure.

However, more *in vitro *and *in vivo *tests in animal models are needed in order to confirm which of the antibiotic regimens used in sepsis may exhibit the most potent anti-inflammatory effect through the highest *in vivo *inhibitory effect against PAF activities and biosynthesis, with simultaneously induction of PAF-degradation, in an effort to increase our understanding of the clinical implications of PAF inhibition with regard to septic shock, severe sepsis and induced multiple organ failure. In another point of view, the simultaneous co-administration of antibiotic regimens with specific PAF antagonists/drugs and/or recombinant PAF-AH should also be considered and may augment the efficacy of antibiotic treatment of sepsis.

The present study is the first step in this direction, while combined with the outcomes of the future in vivo studies it may optimize the efficacy of antibiotic treatment in inflammatory septic conditions.

## Competing interests

The authors declare that they have no competing interests.

## Authors' contributions

ABT conceived of the study, participated in its design and coordination, carried out the *in vitro *studies including the biological test in rabbit platelets, the separation of cells and plasma from rabbit blood, PAF-metabolic enzymes tests, and drafted the manuscript. MC participated in the design of the study. AL participated in the design of the study. GT participated in the design of the study. NT participated in the design of the study and helped to draft the manuscript. CAD conceived of the study, participated in its design and coordination and helped to draft the manuscript. MCL conceived of the study and participated in its design and coordination. All authors have read and approved the final manuscript.
